# Development of an image-based Random Forest classifier for prediction of surgery duration of laparoscopic sigmoid resections

**DOI:** 10.1007/s00384-024-04593-z

**Published:** 2024-01-25

**Authors:** Florian Lippenberger, Sebastian Ziegelmayer, Maximilian Berlet, Hubertus Feussner, Marcus Makowski, Philipp-Alexander Neumann, Markus Graf, Georgios Kaissis, Dirk Wilhelm, Rickmer Braren, Stefan Reischl

**Affiliations:** 1 Institute of Diagnostic and Interventional Radiology, School of Medicine and Health, Klinikum rechts der Isar, Technical University of Munich, Munich, Germany; 2 Department of Surgery, School of Medicine, Technical University of Munich, Ismaninger Straße 22, 81675 Munich, Germany; 3 Research Group MITI, Klinikum rechts der Isar, Technical University Munich, Munich, Germany; 4 Institute for Artificial Intelligence in Medicine and Healthcare, School of Medicine and Faculty of Informatics, Technical University of Munich, Munich, Germany; 5 German Cancer Consortium (DKTK, Partner Site Munich) and German Cancer Research Center, DKFZ, Heidelberg, Germany

**Keywords:** Laparoscopic surgery, Diverticulitis, Computed tomography, Machine learning, Random Forest, Surgery scheduling

## Abstract

**Purpose:**

Sigmoid diverticulitis is a disease with a high socioeconomic burden, accounting for a high number of left-sided colonic resections worldwide. Modern surgical scheduling relies on accurate prediction of operation times to enhance patient care and optimize healthcare resources. This study aims to develop a predictive model for surgery duration in laparoscopic sigmoid resections, based on preoperative CT biometric and demographic patient data.

**Methods:**

This retrospective single-center cohort study included 85 patients who underwent laparoscopic sigmoid resection for diverticular disease. Potentially relevant procedure-specific anatomical parameters recommended by a surgical expert were measured in preoperative CT imaging. After random split into training and test set (75% / 25%) multiclass logistic regression was performed and a Random Forest classifier was trained on CT imaging parameters, patient age, and sex in the training cohort to predict categorized surgery duration. The models were evaluated in the test cohort using established performance metrics including receiver operating characteristics area under the curve (AUROC).

**Results:**

The Random Forest model achieved a good average AUROC of 0.78. It allowed a very good prediction of long (AUROC = 0.89; specificity 0.71; sensitivity 1.0) and short (AUROC = 0.81; specificity 0.77; sensitivity 0.56) procedures. It clearly outperformed the multiclass logistic regression model (AUROC: average = 0.33; short = 0.31; long = 0.22).

**Conclusion:**

A Random Forest classifier trained on demographic and CT imaging biometric patient data could predict procedure duration outliers of laparoscopic sigmoid resections. Pending validation in a multicenter study, this approach could potentially improve procedure scheduling in visceral surgery and be scaled to other procedures.

**Supplementary Information:**

The online version contains supplementary material available at 10.1007/s00384-024-04593-z.

## Introduction

Sigmoid diverticulitis is a common gastroenterological pathology of the Western world with a high socioeconomic burden [1, 2]. Predominantly relying on imaging findings, sophisticated guidelines have been established to triage patients between the available treatment options among which antibiotic treatment, interventional drainage, and emergent surgery are cornerstones [3]. Although resections for diverticular disease have slightly decreased between 2012 and 2017, still a high number of about 27,000 left-sided colonic resections are performed annually due to sigmoid diverticulitis in Germany [4]. Laparoscopic resections have become the gold standard, as they are superior to open surgical procedures regarding major complication rates, pain, quality of life, and hospitalization at the cost of a longer operating time [5–7]. Treatment in high-volume centers is recommended for abdominal surgery as lower complication rates have been shown nationwide for various abdominal procedures [8, 9]. Although centralization could slightly standardize operation times, the variability of the procedure duration may still be high.

Efficient surgical scheduling is a cornerstone but also a challenge of modern healthcare to ensure optimal resource allocation, enhanced patient care, and overall healthcare system effectiveness. Timely and precise surgical scheduling is particularly critical in minimizing patient discomfort and maximizing resource utilization, such as anesthesia services.

To mitigate delays is highly interesting for all surgical disciplines. Having methods to predict the duration of surgical procedures could contribute to the optimization of surgical scheduling and efficient utilization of capacities, as surgery is a costly and demanding aspect of patient care [10, 11]. Although elective surgical scheduling is based on concatenating the most likely time spans for various planned procedures [12], many investigations have attempted to reduce expected errors or improve the timing estimations by using intraoperative data linked to the surgical procedures themselves [13–15]. Indeed, in recent years, there has been a remarkable increase in the development of predictive models for a diverse range of surgical procedures, for instance in orthopedic, otorhinolaryngologic, and neurosurgical procedures [13, 16–20]. These models, often harnessing the power of artificial intelligence and machine learning, have demonstrated their potential for healthcare administration. By tailoring predictions to the unique characteristics of each procedure, they offer an interesting tool for surgical scheduling, and thus resource allocation and patient care optimization. Although predicting surgical procedures has been demonstrated for the previously named disciplines, no approaches have been published for visceral surgery.

Beyond simple regression methods for predicting operating room times from scratch based on patient-specific data, more in-depth surgical duration prediction techniques, especially using machine learning have been reported. Random Forests have been proven useful for prediction tasks in surgery, such as possible procedure-related complications [21–23]. This ensemble learning method basically aggregates the decisions of a self-created collection of upon a given dataset of randomly assembled decision trees by bootstrapping and bagging, which altogether allow more exact assumptions than simple decision trees for classification tasks [21]. As Random Forests are computationally rather inexpensive while still allowing precise predictions [24], their training and further implementation in the day-to-day hospital workflow can be performed quickly and efficiently.

Requirements for easy applicable models are a limited number and easy acquirable input variables to keep the effort for the user as low as possible. Patient anatomical features as spatial prerequisites for surgery and demographic patient data might allow for predictions of surgery duration or even identification of outliers in surgery time since anatomical variety has been shown to have an impact on operative time [25]. In cases of diverticular disease, the extent of the disease can easily be determined through preoperative CT scans, as well as other anatomical features of the patient that could potentially influence the time required for laparoscopic procedures. Therefore, it was selected as a model disease for this proof-of-principle study.

The aim of this study is to develop and evaluate a model on radiologically determined anatomical distances, as well as basic demographic patient data, to predict the duration of surgery of laparoscopic sigmoid resections.

## Material and methods

### Study design and patient collective

This single-center retrospective cohort study was approved by the local ethics committee (no. 10/19S). Patients who underwent sigmoid resection for diverticulitis at our institution between April 2009 and October 2020 were identified from a prospectively curated database of all patients undergoing colorectal resections at our center. Patients were excluded for (1) lack of preoperative CT scan, (2) open or robotic-assisted procedures, (3) preoperatively intended simultaneous operative procedures, and (4) prior abdominal surgery.

### Imaging parameter acquisition

For all included patients, various measurements were taken from the most recent CT scan prior to resection. A board-certified visceral surgeon with 20 years of experience and specialization in oncological and non-oncological colorectal surgery suggested potentially relevant anatomical distances that could affect surgery duration based on clinical experience and recent literature: “spleen-colic flexure,” “colic flexure-inflammation,” “promontory-symphysis,” “subcutaneous fat,” and “gluteal fat” (Supplemental Table 1, Supplemental Fig. 1).

**Table 1:** Patient cohort and surgery duration subgroups

	**Total (*n* = 85)**	**“Short” (≤ 150 min) (*n* = 28)**	**“Intermediate” (150–199 min) (*n* = 28)**	**“Long” (≥ 200 min) (*n* = 29)**	***P***
Sex					**< *0.05**** / 0.48 ****/* < *0.05***
Male	46	8	17	21	
Female	39	20	11	8	
Stage (CDD)					*0.5 / 0.5 / 0.99*
Type 2 (a/b/c)	20 (18/1/1)	8 (8/0/0)	5 (5/0/0)	7 (5/1/1)	
Type 3 (a/b/c)	65 (1/55/9)	20 (1/16/3)	23 (0/21/2)	22 (0/18/4)	
Age (in years)	54.9 ± 11.4	55.5 ± 11.0	57.0 ± 11.9	52.2 ± 11.1	*0.26*
“spleen–colic flexure” (in mm)	68.4 ± 24.8	62.7 ± 23.3	67.6 ± 26.8	74.7 ± 23.5	*0.18*
“colic flexure–inflammation” (in mm)	209.7 ± 47.6	219.7 ± 35.8	198.6 ± 48.8	210.8 ± 55.1	*0.25*
“promontory – symphysis” (in mm)	115.3 ± 11.9	121.4 ± 10.0	110.9 ± 12.1	113.8 ± 11.4	**< *0.05***
“subcutaneous fat” (in mm)	26.0 ± 11.5	24.6 ± 10.2	23.3 ± 9.1	29.9 ± 13.7	*0.07*
“gluteal fat” (in mm)	26.8 ± 10.2	28.5 ± 11.0	24.3 ± 9.9	27.6 ± 9.7	*0.28*
Surgery duration (in min)	178.8 ± 46.6	130.1 ± 13.0	171.4 ± 13.6	232.8 ± 26.9	**< *0.01***

Parametric data are presented as mean with standard deviation. P values below the significance level of 0.05 are marked in bold. Abbreviations: CDD = Classification of diverticular disease.

**Fig. 1:**
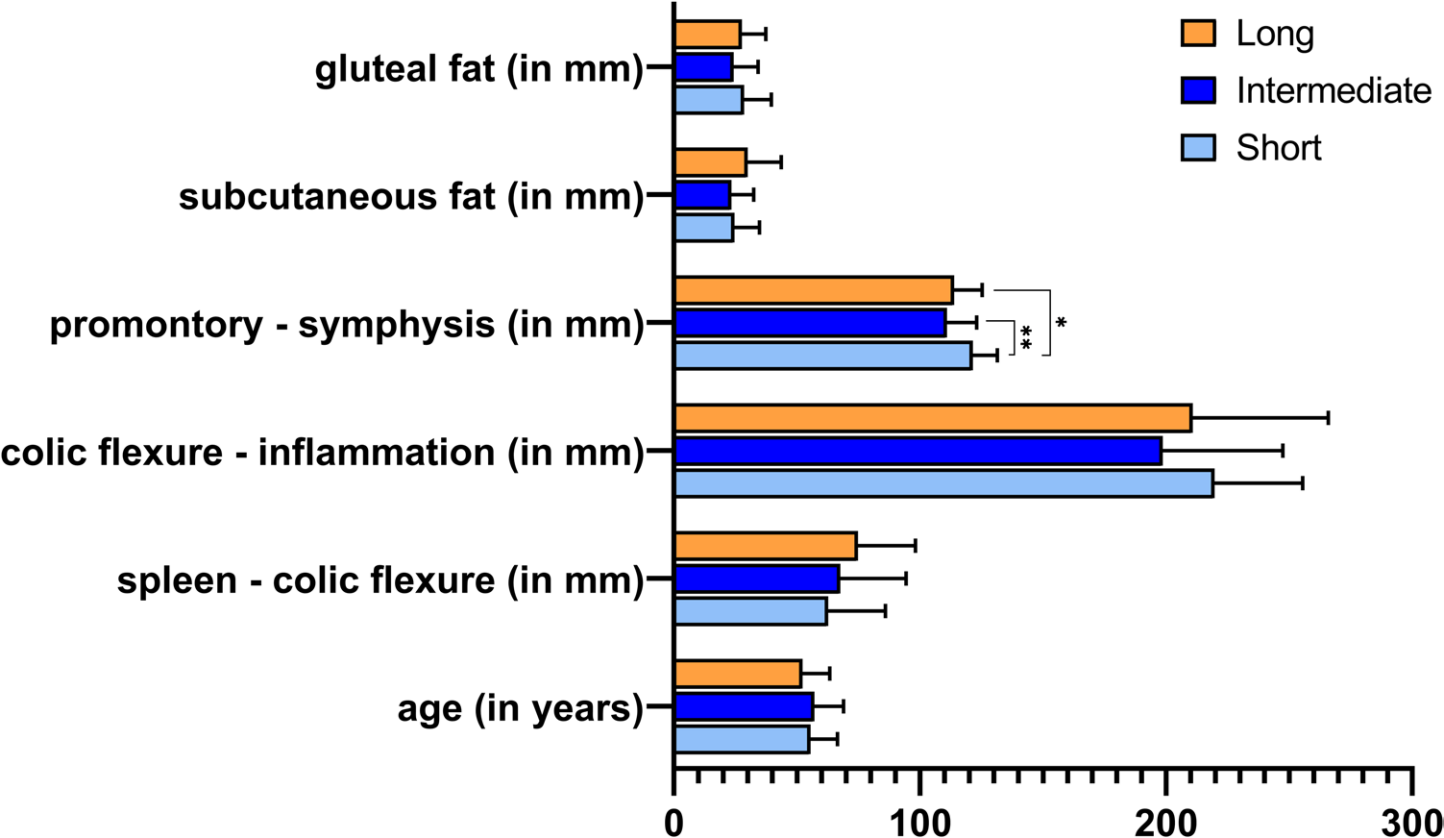
Anatomical distances compared between the surgery duration classes. Bar plots depicting the distribution of the anatomic features and age. Data are presented as mean values and standard deviation. Significant differences are indicated as *p < 0.05, **p < 0.01

### Patient and procedure-related data

Patient age, sex, Classification of Diverticular Disease (CDD) stage, and performing surgeon were extracted from the electronic patient data management system IS-H (SAP, Germany). As the primary outcome of this study, surgery duration was defined as the time from the first skin incision to completion of the skin suture as documented in the electronic patient database. Theater nurses perform documentation of procedure time simultaneously to the operation. Age and sex were selected as additional input variables, as they are very easy to collect and readily available for all patients.

### Statistical analysis

All statistical analyses were calculated in Python (scipy package 1.7.3). Normal distribution was investigated by the Shapiro-Wilk test. Statistical differences were determined for parametrical data by ANOVA with the Bonferroni post-hoc test or unpaired *t*-test for independent samples, respectively. Class rank correlations between imaging parameters and surgery duration were investigated with Spearman’s *ρ*. In this study, a significance level of α = 0.05 was employed.

### Multiclass logistic regression analyses

The dependent variable “surgery duration” was discretized by class size balanced trichotomization into an ordinal categorical variable to avoid the class imbalance problem during further analyses and as there are no evident intrinsic or extrinsic cut-offs. The independent variables were the five imaging parameters (in mm), patient age (in years), and sex (male/female). Preprocessing of the training data included One-Hot-Encoding for patient sex and standard scaling for all other variables. Subsequently, the cohort was randomly split stratified by label into a training set (75% of the study population, *n* = 63) and a test set (25% of the study population, *n* = 22). Multiclass logistic regression of the dependent variable “surgery duration” was performed in the training set while tested in the test set. For visualization, confusion matrices were generated, and specificity, sensitivity, and positive and negative predictive values were calculated at a decision threshold of 0.5.

### Training and testing of Random Forest classifier

For the training, testing, and evaluation of the Random Forest model, Python with the scikit-learn 1.0.2 package was used. The identical dataset split as in the logistic regression model was used. We used 100 decision trees with a maximum of 11 nodes and the decision criterion “entropy.” The model was then fit to the three surgery duration classes. Lastly, we evaluated the predictive performance by plotting receiver operating characteristic (ROC) curves for multiple classes. For evaluation, each class was compared to the two remaining classes taken together. Simultaneously, the area under the curve (AUROC) was calculated for each class, as well as a macro-average and a micro-average of all three, which takes the proportion of the classes in the testing set into account. Additionally, confusion matrices of the test set were plotted, and accuracy, specificity, sensitivity, and positive and negative predictive values were calculated.

## Results

### Patient cohort and surgery indication

Eighty-five patients (46 male, 39 female) with a mean age of 54.9 ± 11.4 years who had undergone an abdominal CT between April 2009 and October 2020, which revealed signs of diverticulitis prior to undergoing laparoscopic sigmoid resection, were included in the study (Table 1). Surgical indications were mostly relapsing diverticulitis without complications (CDD stage 3b, *n* = 55, 65%) and acute complicated diverticulitis with microabscess (< 1 cm, CDD stage 2a, *n* = 18, 21%), which accounted for 85.8% of the indications in total. The surgical procedures took on average 178.8 ± 46.6 min. Surgery times did not significantly differ between CDD stages 2 and 3 (*p* = 0.99). Within the subgroups, “short” surgeries took equal or less than 150 min, “intermediate” surgeries were those with durations between 150 and 200 min, and “long” surgeries were those that took equal or more than 200 min.

### Univariate correlation of imaging parameters and surgery duration

The only distance that showed a significant difference between the duration groups was “promontory–symphysis” (*p* < 0.05). This parameter was significantly inversely correlated with the rank of surgery duration classes (Spearman’s *ρ* = −0.27, *p* < 0.01). All other anatomical distances did not show significant differences between the surgery duration groups (see Fig. 1 and Table 1).

### Subgroup analysis for performing surgeon

Overall, 20 different surgeons performed the included procedures. The five surgeons who performed at least five documented procedures in the cohort accounted for 53 out of the 85 resections (64.2%). The surgery durations ranged from 163 to 226 min, with a standard deviation of 18.5 min between their average surgery durations. The median surgery duration did neither differ significantly between the surgeons (*p* = 0.14) nor between the five surgeons that performed the most procedures (*p* = 0.34) (Supplementary Fig. 2).

### Multiclass logistic regression analyses

**Fig. 2:**
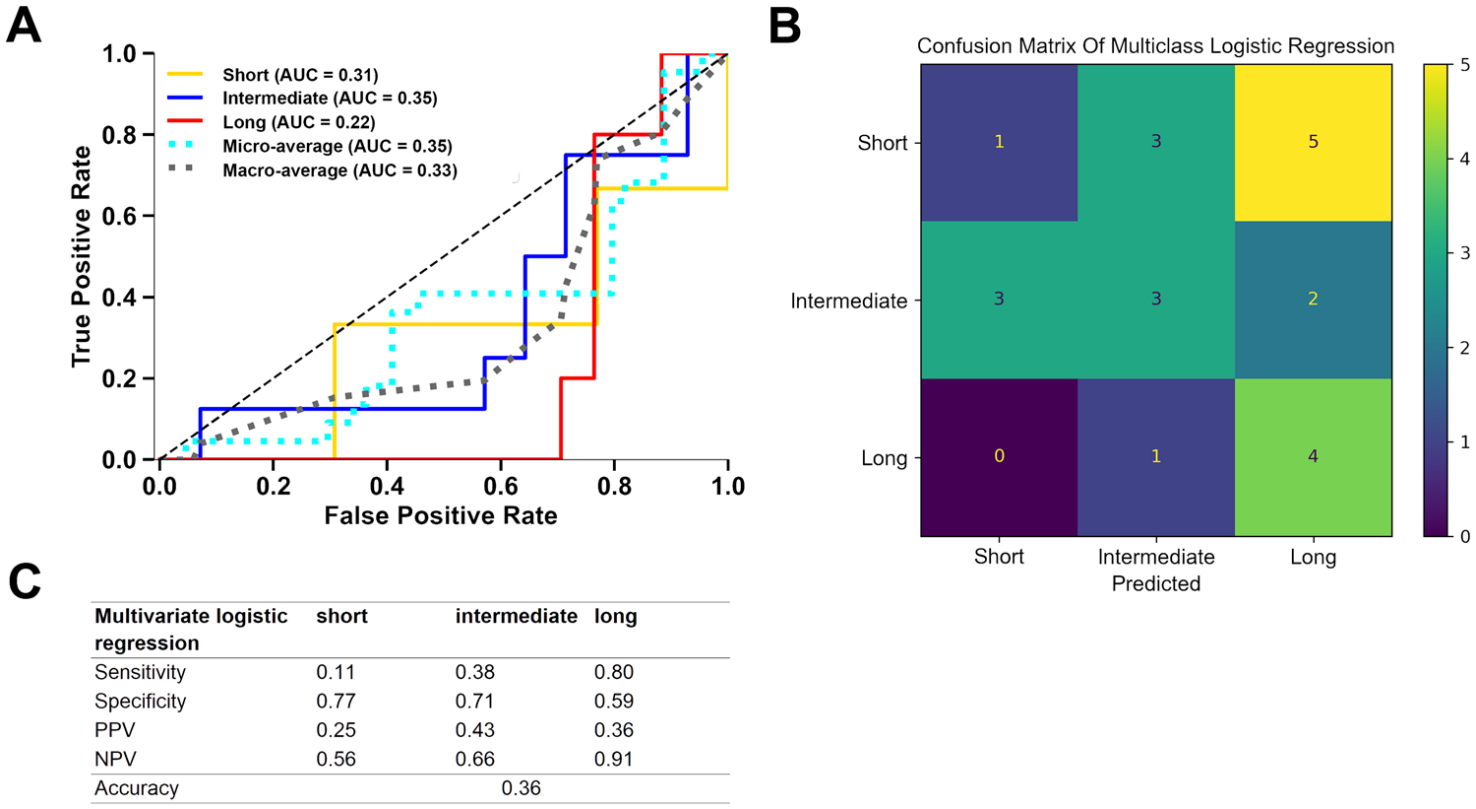
Performance metrics of the multiclass logistic regression model. **A** Receiver operating characteristics, **B** confusion matrix, and **C** sensitivity, specificity, positive predictive value (PPV), negative predictive values (NPV), and accuracy for a model decision threshold of 0.5 are shown

Multiclass logistic regression for the dependent variable “surgery duration” was performed and tested as described in the "Material and methods" section. After random split, the training and the testing dataset did not differ in any of the independent input variables or surgery duration as dependent variable (Supplemental Table 2). The logistic regression model achieved macro-average AUROC values of 0.33 (Fig. 2). In detail, it achieved an AUROC value of 0.22 for long, 0.35 for intermediate, and 0.31 for short procedures. The accuracy of the logistic regression model was 0.36. At a model threshold of 0.5, the specificity for “long”’ procedures was 0.59 at a sensitivity of 0.80.

### Random Forest classifier model performance

**Fig. 3:**
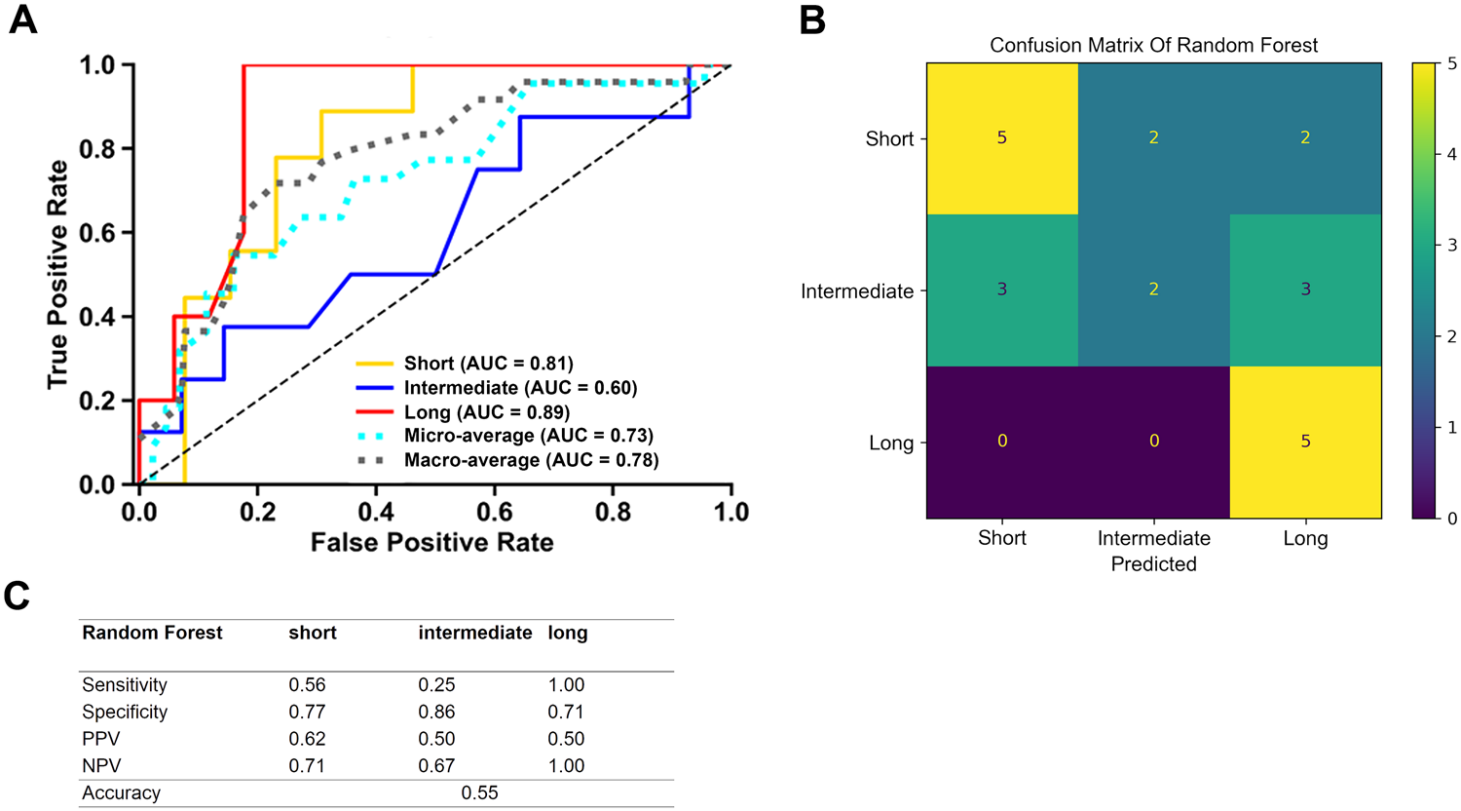
Performance metrics of the Random Forest model. **A** Receiver operating characteristics, **B** confusion matrix, and **C** sensitivity, specificity, positive predictive value (PPV), negative predictive values (NPV), and accuracy are shown

Additionally, we used a Random Forest architecture to potentially improve the prediction. The same training and testing set split as in the multiclass logistic regression model was used. Trained on all parameters (imaging parameters, age, sex), the Random Forest classifier achieved good overall AUROC values (macro-average of 0.78, micro-average of 0.73, see Fig. 3), which outperformed the logistic regression model. Particularly, long surgery durations could be predicted with an excellent AUROC of 0.89. Additionally, short procedures were predicted with an excellent AUC of 0.81, while discrimination was worst for intermediate surgery duration (AUROC of 0.60). Likewise, the accuracy of the Random Forest at 0.55 outperformed the logistic regression model, and particularly for long procedures, the model had a very good sensitivity of 1.0 at a moderate specificity of 0.71.

## Discussion

In this study, we developed a machine learning model, capable of predicting the surgery duration for laparoscopic sigmoidectomy procedures in case of diverticular disease. By incorporating easily accessible demographic patient data and anatomic distances from preoperative CT scans, our model forecasts the amount of time required for the surgical procedure with a good performance, particularly for upper outliers in surgery duration.

The decision to include the seven independent variables was made due to their general availability and simplicity of data collection in order to develop an efficient tool for surgical scheduling. We performed a step-by-step approach with increasing methodological complexity, from simple univariate correlations to multiple linear and multiclass logistic regression and finally a Random Forest to generate a model capable of predicting outliers in surgery duration.

Only “promontory–symphysis” showed an inverse correlation with surgery duration in the Spearman correlation that can be explained by facilitated handling of laparoscopic instruments within the pelvis for resection and anastomosis formation. While Targarona et al. previously presented pelvic diameter as an influence on laparoscopic rectal surgery, this factor has not been investigated for benign laparoscopic sigmoid surgery [25]. Likewise, the logistic regression model showed a weak performance with a macro-average AUROC of 0.33 and an accuracy of 0.36 to predict surgery duration, why the decision for the Random Forest model was taken.

Despite being trained on independent variables that were individually predominantly non-significant, the Random Forest model achieved a very good AUROC of 0.89 to predict “long” procedures, while also reaching a very good prediction for “short” procedures (AUROC 0.81), which outperformed the multiclass logistic regression model. Solid prediction was additionally reflected by the accuracy of 0.55. Interestingly, without threshold calibration, the model had a sensitivity for “long” procedures of 1.0 at a specificity of 0.71, which seems to be practicable as long outliers should not be missed, but overdiagnosis is a lesser concern.

Accurate prediction of surgery duration is crucial for scheduling surgical procedures [15], and our model’s ability to do so can lead to improvements in operating room utilization, staffing levels and resource allocation, resulting in cost savings and increased efficiency. Upper limit outliers in operation time are particularly problematic for the smooth sequencing of daily surgical programs, as anesthesia can only be supplied for a certain maximum time frame. On the other hand, shorter-than-average procedures can be filled with on-call surgeries, especially in hospitals with high patient volumes. Our model can be easily implemented as a decision-making tool for surgical scheduling, as it requires simple measurements and basic demographic patient data that are readily available.

Only one study has reported the prediction of surgery time for a distinct procedure, which was hip arthroplasty. Despite being trained on about 1200 cases, it achieved only comparable AUROC values to our study, presumably due to individually less relevant independent variables (mostly procedure-related and demographic patient data) [17, 18]. Other studies have examined far broader procedure collectives. For example, one study examined the predictability of surgery duration for various otorhinolaryngologic procedures using different ensemble learning methods and achieved good results [20]. However, this study included mostly procedure, institution, and infrastructure data, with only age and little data on comorbidities in their model. Another study demonstrated that even orthopedic procedure durations can be predicted by ensemble learning methods [26]. In general, Random Forest models and gradient-boosted regression trees have shown the best performance in different classification tasks for surgery times in different fields [16, 17, 20, 26, 27].

Our study has several limitations that should be discussed in the following. Foremost among these limitations is the relatively small sample size. However, it is important to note that our study included 85 patients over the study period, which significantly exceeds the nationwide average. Additionally, the limited number of primary surgeons is an important limitation, as the performing surgeon may be an important confounder in our study. Still, in sensitivity analysis, we could not find any difference in surgery times between the surgeons. Despite this, the constrained size of our patient cohort limited our ability to employ state-of-the-art techniques such as the prediction of surgical times from full-volume abdominal CT scans using convolutional neural networks (CNNs). Despite the limitations, our model demonstrated a good performance. This may be attributed to the selection of relevant, patient-specific anatomical and demographic data included in our model, as well as the focus on a single surgical procedure. However, it should be noted that our model lacks generalizability to other abdominal surgery procedures. In summary, the project requires careful interpretation and underscores the imperative for future multicenter studies with larger patient population to enhance external validity and facilitate the use of cutting-edge approaches. Furthermore, the transformation of a continuous variable into a categorical variable, in the case of our study trichotomization bears some important risks [28]. However, we can provide a rationale for trichotomization as the intermediate class is the expected class, while one other class was defined for longer or shorter procedures to be able to adapt the expected time in scheduling. Lastly, the model was developed and evaluated on a specific population and may not necessarily be applicable to other patient populations with different demographics, comorbidities, or anatomical variations.

## Conclusion

In this proof-of-concept study, we trained a Random Forest model able to predict outliers in surgery duration of laparoscopic sigmoid resections for diverticular disease on easily accessible data from preoperative abdominal CT scans and basic patient information with good accuracy. Although a multicenter study is required to guarantee external validity, this approach could be implemented into clinical routine to optimize surgical scheduling.

## Supplementary information

Below is the link to the electronic supplementary material.

Supplementary file1 (DOCX 299 KB)

## Data Availability

The data that support the findings of this study are not openly available due to reasons of sensitivity and are available from the corresponding author upon reasonable request.
