# Supplementary material for: Development of an image-based Random Forest classifier for prediction of surgery duration of laparoscopic sigmoid resections
Source: Int J Colorectal Dis. 2024 Jan 25;39(1):21. doi: 10.1007/s00384-024-04593-z (PMC10811180; doi:10.1007/s00384-024-04593-z)
Supplement: Supplementary file 1 — Supplementary file1 (DOCX 299 KB) [file 384_2024_4593_MOESM1_ESM.docx]

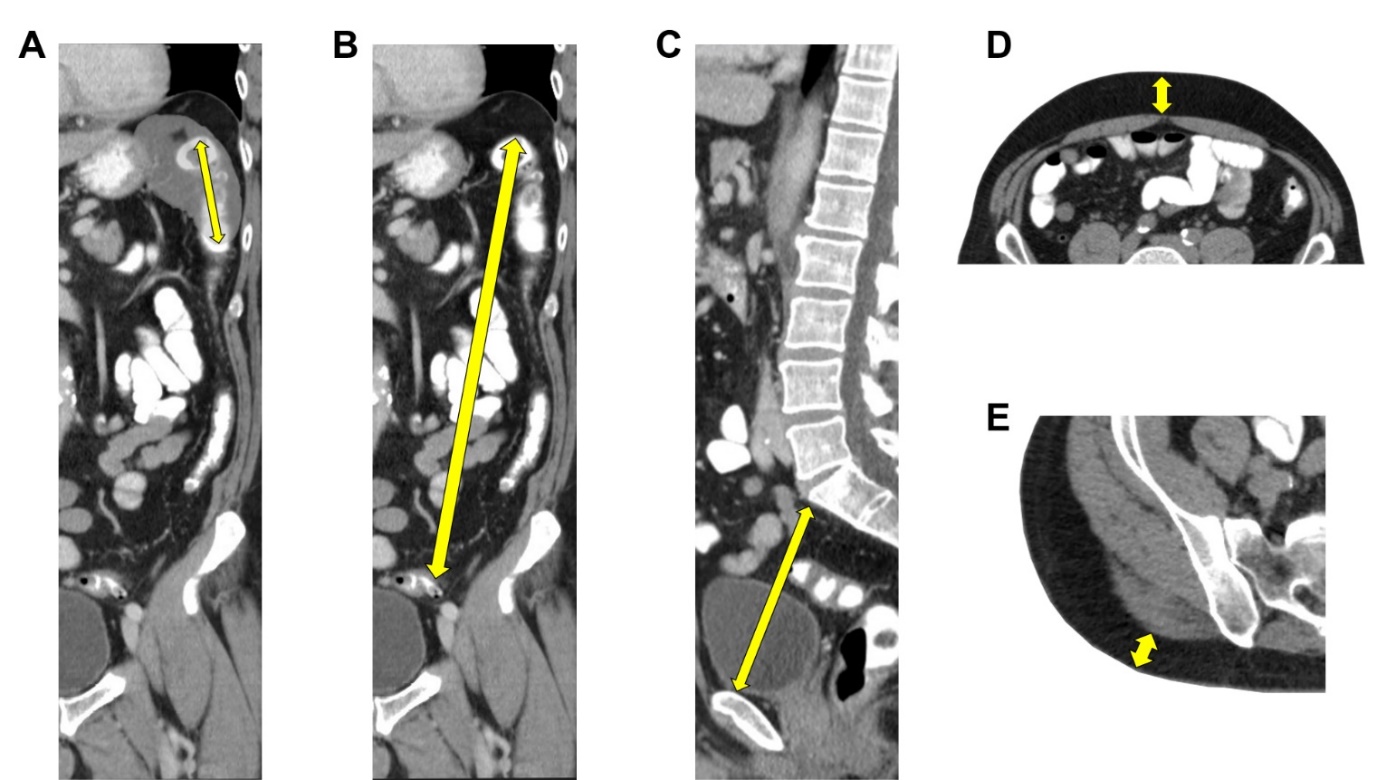


**Supplemental figure 1. Measurement of the potentially relevant anatomical distances in most recent preoperative CT scans.** (A) 'spleen - colic flexure', (B) 'colic flexure - inflammation', (C) 'promontory - symphysis', (D) 'subcutaneus fat' and (E) 'gluteal fat' were determined as potentially relevant features for training of the Random Forest classifier.

**Supplemental table 1**. Expert-opinion potentially relevant disease-specific anatomical distances determined in preoperative CT scans.

| **Anatomical distance** | **Indicates** | **Potentially influences** | **Measurement plane** | **Measurement description** |
| --- | --- | --- | --- | --- |
| (A) 'spleen - colic flexure' | general site narrowness | resection and anastomosis formation | coronal | Most cranial point of left colic flexure to most caudal point of lower splenic pole |
| (B)  'colic flexure - inflammation' | colon transposition extent | resection and anastomosis formation | coronal | Most cranial point of left colic flexure to oral inflammation boundary |
| (C)  'promontory - symphysis' | pelvic site narrowness | resection and anastomosis formation | median | Promontory to most posterior part of pubic symphysis |
| (D)  'subcutaneous fat' | obesity | laparoscopic approach | axial | Skin surface to abdominal fascial in the median line |
| (E)  'gluteal fat' | obesity | laparoscopic approach | axial | Skin surface to fascia of greater gluteal muscle at S1 |

**Supplemental table 2.** Relevant characteristics of the total cohort and the training and test subset are shown.

|  | **Total**  **(n = 85)** | **Training subset (n = 63)** | **Testing subset  (n = 22)** | ***P*** |
| --- | --- | --- | --- | --- |
| Sex  male  female | 46 (100 %)  39 (100 %) | 34 (74 %)  29 (74 %) | 12 (26 %)  10 (26 %) | 0.98  0.97 |
| CDD Stage  Stage 2  Stage 3 | 20 (18/1/1) 65 (1/55/9) | 12 (10/1/1)  51 (1/45/5) | 8 (8/0/0) 14 (0/10/4) | 0.40  0.16 |
| Age (years) | 54.9 ± 11.4 | 55.0 ± 12.0 | 54.1 ± 9.2 | 0.74 |
| 'spleen - colic flexure' (in mm) | 68.4 ± 24.8 | 69.40 ± 22.0 | 65.59 ± 31.8 | 0.53 |
| 'colic flexure - inflammation' (in mm) | 209.7 ± 47.6 | 209.7 ± 46.8 | 209.7 ± 50.9 | 0.99 |
| 'promontory - symphysis' (in mm) | 115.3 ± 11.9 | 114.7 ± 12.2 | 117.1 ± 11.1 | 0.43 |
| 'subcutaneous fat' (in mm) | 26.0 ± 11.5 | 25.52 ± 11.8 | 27.3 ± 10.5 | 0.55 |
| 'gluteal fat' (in mm) | 26.8 ± 10.2 | 25.9 ± 9.9 | 29.3 ± 10.9 | 0.18 |
| Surgery duration (in minutes) | 178.8 ± 46.6 | 181.4 ± 45.0 | 171.2 ± 51.2 | 0.38 |


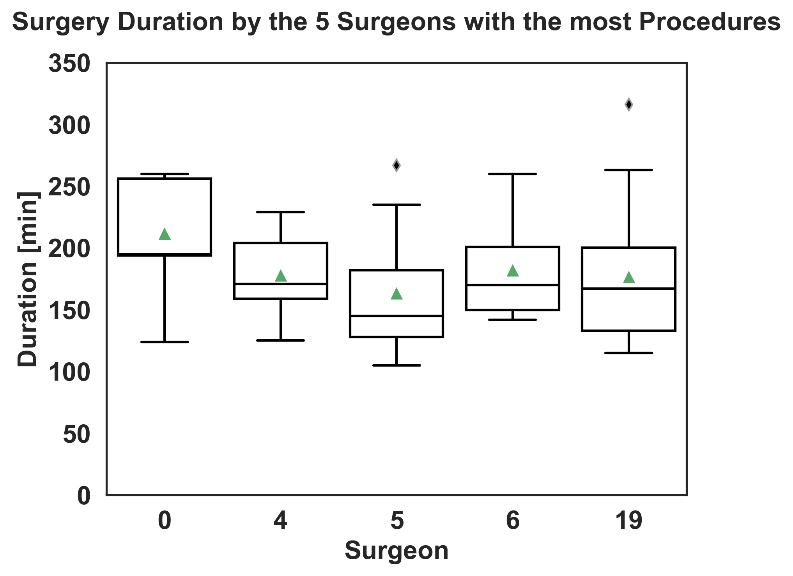


**Supplemental figure 2. Surgery duration of the five surgeons with the most procedures.** There was no difference between the surgery times of all included surgeons (p = 0.14), nor between the five surgeons with the most procedures (p = 0.34; 64 % of procedures). Data is presented as median (bar) / mean (triangle) surgery duration with quartiles (box) and range (whiskers) as well as outliers (diamonds) for the five surgeons with the most surgeries. Statistical differences between the surgeons were examined by one-way ANOVA.

| **Section/Topic** | **Item** |  | **Checklist Item** | **Page** |
| --- | --- | --- | --- | --- |
| **Title and abstract** | | | | |
| Title | 1 | D;V | Identify the study as developing and/or validating a multivariable prediction model, the target population, and the outcome to be predicted. | 1 |
| Abstract | 2 | D;V | Provide a summary of objectives, study design, setting, participants, sample size, predictors, outcome, statistical analysis, results, and conclusions. | 2 |
| **Introduction** | | | | |
| Background and objectives | 3a | D;V | Explain the medical context (including whether diagnostic or prognostic) and rationale for developing or validating the multivariable prediction model, including references to existing models. | 3,4 |
|  | 3b | D;V | Specify the objectives, including whether the study describes the development or validation of the model or both. | 4 |
| **Methods** | | | | |
| Source of data | 4a | D;V | Describe the study design or source of data (e.g., randomized trial, cohort, or registry data), separately for the development and validation data sets, if applicable. | 5 |
|  | 4b | D;V | Specify the key study dates, including start of accrual; end of accrual; and, if applicable, end of follow-up. | 5 |
| Participants | 5a | D;V | Specify key elements of the study setting (e.g., primary care, secondary care, general population) including number and location of centres. | 5 |
|  | 5b | D;V | Describe eligibility criteria for participants. | 5 |
|  | 5c | D;V | Give details of treatments received, if relevant. | 7 |
| Outcome | 6a | D;V | Clearly define the outcome that is predicted by the prediction model, including how and when assessed. | 5, 6, 7 |
|  | 6b | D;V | Report any actions to blind assessment of the outcome to be predicted. | retrospective study |
| Predictors | 7a | D;V | Clearly define all predictors used in developing or validating the multivariable prediction model, including how and when they were measured. | 5, 6 |
|  | 7b | D;V | Report any actions to blind assessment of predictors for the outcome and other predictors. | retrospective study |
| Sample size | 8 | D;V | Explain how the study size was arrived at. | 5 |
| Missing data | 9 | D;V | Describe how missing data were handled (e.g., complete-case analysis, single imputation, multiple imputation) with details of any imputation method. | no missing input data |
| Statistical analysis methods | 10a | D | Describe how predictors were handled in the analyses. | 5 , 6 , 7 |
|  | 10b | D | Specify type of model, all model-building procedures (including any predictor selection), and method for internal validation. | 5, 6, 7 |
|  | 10c | V | For validation, describe how the predictions were calculated. | 5, 6, 7 |
|  | 10d | D;V | Specify all measures used to assess model performance and, if relevant, to compare multiple models. | 6, 7 |
|  | 10e | V | Describe any model updating (e.g., recalibration) arising from the validation, if done. | not performed |
| Risk groups | 11 | D;V | Provide details on how risk groups were created, if done. | not performed |
| Development vs. validation | 12 | V | For validation, identify any differences from the development data in setting, eligibility criteria, outcome, and predictors. | Suppl. table 1 |
| **Results** | | | | |
| Participants | 13a | D;V | Describe the flow of participants through the study, including the number of participants with and without the outcome and, if applicable, a summary of the follow-up time. A diagram may be helpful. | 7, 8, Fig. 1 |
|  | 13b | D;V | Describe the characteristics of the participants (basic demographics, clinical features, available predictors), including the number of participants with missing data for predictors and outcome. | 7, 8, Table 2 |
|  | 13c | V | For validation, show a comparison with the development data of the distribution of important variables (demographics, predictors and outcome). | Suppl. table 1 |
| Model development | 14a | D | Specify the number of participants and outcome events in each analysis. | 7, 8, Table 1&2 |
|  | 14b | D | If done, report the unadjusted association between each candidate predictor and outcome. | 7, 8 |
| Model specification | 15a | D | Present the full prediction model to allow predictions for individuals (i.e., all regression coefficients, and model intercept or baseline survival at a given time point). | code available upon request |
|  | 15b | D | Explain how to the use the prediction model. | see 15a |
| Model performance | 16 | D;V | Report performance measures (with CIs) for the prediction model. | 7, 8, Fig. 4 and 5 |
| Model-updating | 17 | V | If done, report the results from any model updating (i.e., model specification, model performance). | - |
| **Discussion** | | | | |
| Limitations | 18 | D;V | Discuss any limitations of the study (such as nonrepresentative sample, few events per predictor, missing data). | 10,11 |
| Interpretation | 19a | V | For validation, discuss the results with reference to performance in the development data, and any other validation data. | 9, 10, 11 |
|  | 19b | D;V | Give an overall interpretation of the results, considering objectives, limitations, results from similar studies, and other relevant evidence. | 9, 10 |
| Implications | 20 | D;V | Discuss the potential clinical use of the model and implications for future research. | 9, 10 |
| **Other information** | | | | |
| Supplementary information | 21 | D;V | Provide information about the availability of supplementary resources, such as study protocol, Web calculator, and data sets. | 11 |
| Funding | 22 | D;V | Give the source of funding and the role of the funders for the present study. | 11 |


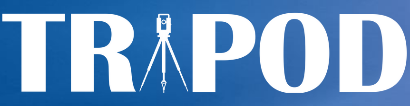
*Items relevant only to the development of a prediction model are denoted by D, items relating solely to a validation of a prediction model are denoted by V, and items relating to both are denoted D;V. We recommend using the TRIPOD Checklist in conjunction with the TRIPOD Explanation and Elaboration document.
